# Management practices impact vine carbohydrate status to a greater extent than vine productivity

**DOI:** 10.3389/fpls.2014.00283

**Published:** 2014-06-27

**Authors:** Anne Pellegrino, Peter Clingeleffer, Nicola Cooley, Rob Walker

**Affiliations:** ^1^CSIRO Plant IndustryGlen Osmond, SA, Australia; ^2^Montpellier SupAgro, UMR LEPSEMontpellier, France; ^3^Higher Education - Primary Industries, Northern Melbourne Institute of TAFEMelbourne, VIC, Australia

**Keywords:** grapevine, pruning, deficit irrigation, carbohydrates, vine physiology, variety

## Abstract

Light pruning and deficit irrigation regimes are practices which are widely used in high yielding commercial vineyards in the warm climate regions of Australia. Little information is available on their impacts on carbohydrate dynamics in vegetative organs within and between seasons, and on the resulting plant capacity to maintain productivity and ripen fruits. This study was conducted to address this gap in knowledge over five vintages on *Vitis vinifera* L. cv. Cabernet Franc, Shiraz, and Cabernet Sauvignon in the Sunraysia region of Victoria, Australia. Lighter pruning did not change the total carbohydrates concentration and composition in wood and roots within seasons in Cabernet Franc and Shiraz. However, the total carbohydrate pool (starch and soluble sugars) at the end of dormancy increased under lighter pruning, due to higher vine size, associated with retention and growth of old-wood (trunk and cordons). Water deficit negatively impacted trunk and leaf starch concentrations, over the day and within seasons in Cabernet Sauvignon. Soluble sugars concentrations in these tissues tended to be higher under limited water supply, possibly due to higher sugar mobilization as photosynthesis decreased. Trunk carbohydrate concentrations markedly varied within and between seasons, highlighting the importance of interactive factors such as crop load and climate on carbon status. The period between fruit-set and véraison was shown to be critical for its impact on the balance between carbon accretion and depletion, especially under water deficit. The lower leaf and trunk starch concentration under water deficit resulted in a decrease of yield components at harvest, while similar yields were reached for all pruning systems. The sugar allocated to berries at harvest remained remarkably stable for all practices and seasons, irrespective of vine yield and carbohydrate status in vegetative organs in Shiraz and Cabernet Sauvignon.

## Introduction

In high yielding vineyards grown in warm regions of Australia, there has been widespread adoption of lighter pruning techniques such as mechanical hedging and minimal pruning, together with deficit irrigation techniques to enhance red wine quality (Clingeleffer, 2010). These practices are likely to impact the dynamics of vine carbohydrate status within and between seasons and also vine productivity (yield and sugar content). Within season, vine carbohydrate dynamics rely on the balance between carbon supply from canopy assimilation (leaf area, photosynthesis), and carbon demand, which depends on growth and maintenance of the different sinks (berries, leaves, stems, trunk, and root). The general phenological trend reported in the literature is a mobilization of reserve after budburst to support extensive canopy growth in spring, and hence a progressive reserve restoration starting at flowering or later on, depending on the cultivars, crop load, and pedo-climatic conditions (Bates et al., 2002; Holzapfel and Smith, 2012; Zufferey et al., 2012). The rate of carbon reserves replenishment, together with the timing of the transition between heterotrophic (root) and autotrophic (leaf) carbon allocation mode, were shown to be critical for early reproductive development (Candolfi-Vasconcelos and Koblet, 1990; Zapata et al., 2004; Lebon et al., 2008 and references therein). Starch is a major carbohydrate reserve in grapevine perennial tissues (Weyand and Schultz, 2006; Holzapfel and Smith, 2012; Zufferey et al., 2012). It predominates in roots (Bates et al., 2002), where its maximal concentration in winter reached one third of root dry weight in Pinot Noir, Merlot, and Shiraz (Zapata et al., 2004; Holzapfel and Smith, 2012). Soluble sugars (sucrose, glucose, and fructose) significantly contribute to the total carbohydrate reserves in the above ground tissue. Concentrations up to 7% of dry weight were measured in winter on Shiraz and Chasselas (Holzapfel and Smith, 2012; Zufferey et al., 2012).

Grapevine carbohydrate dynamics and productivity rely on crop load management. Lower carbon supply due to early defoliation (before bloom) reduced berry set and berry mass (Candolfi-Vasconcelos and Koblet, 1990; Palliotti et al., 2011). Vine defoliation at harvest reduced the total carbon reserve in trunk, resulting in fewer inflorescences per shoot, fewer flowers per inflorescences and lower yield in the following seasons (Holzapfel et al., 2006; Holzapfel and Smith, 2012). Reducing crop load and thus carbon demand at the onset of ripening had the reverse effect (Holzapfel and Smith, 2012). Berry ripening (sugar concentration) was also accelerated by defoliation before bloom or by crop removal after fruit set, probably due to lower competition between reproductive and vegetative sinks (Petrie and Clingeleffer, 2006; Palliotti et al., 2011). Climate is a major interactive factor impacting carbohydrate reserve dynamics in perennial tissues in addition to crop load (Holzapfel and Smith, 2012). Trunk soluble carbohydrate concentration was shown to be negatively correlated to mean air temperature during the preceding week in Chasselas (Zufferey et al., 2012). Warm temperatures are likely to negatively impact carbon balance through faster canopy development and enhanced respiration rates, while source activity (assimilation rate) may plateau (Schultz, 2000; Zufferey et al., 2000; Lebon et al., 2004; Pallas et al., 2008). Depending on cultivar behavior, high evaporative demand (VPD) may exacerbate the limitation in carbon availability during summer due to stomatal closure and leaf temperature increase (Soar et al., 2006; Prieto et al., 2010; Rogiers et al., 2012). The post-harvest canopy function should thus be an important consideration for high yielding vineyards grown in a warm and dry climate, in order to provide high levels of stored carbohydrates going into dormancy for sustained crop productivity (Sommer and Clingeleffer, 1996; Smith and Holzapfel, 2009). Under such conditions, pruning practices have the potential to increase the vine carbon status by optimizing the balance between the shoots number and the pool of carbohydrate reserves. Lighter pruning generally leads to a higher proportion of old wood and higher yields (Clingeleffer and Sommer, 1995; Clingeleffer, 2010). The impact of lighter pruning on vine carbohydrate dynamics within and between seasons in a warm climate and on subsequent vine capacity to sustain and mature high crop load is not well-understood.

Regulated deficit irrigation (RDI) is the most common deficit irrigation strategy implemented in warm climate grape-growing regions. This practice is aimed at optimizing the balance between grapevine vigor and potential production. The reduction of irrigation amount after berry set in RDI slows canopy development and decreases berry size (Matthews and Anderson, 1989; Ojeda et al., 2001; Kriedemann and Goodwin, 2004 and references therein). Some source limitations are expected under deficit irrigation due to lower total leaf area, together with lower photosynthesis rate (Lebon et al., 2006). However, the combination of water deficit and high temperatures may cause severe decline in photosynthesis rate, together with an increase in leaf senescence, thus resulting in important reduction in carbohydrate supply (Chaves et al., 2010 and reference therein). Photosynthesis decline under limited water supply was associated with a decrease in leaf starch concentration in Savatiano (Patakas and Noitsakis, 2001). Wood and root starch concentrations were also lower under water deficit in Shiraz (Holzapfel et al., 2010). In contrast, water deficit enhanced root sucrose concentration, thus increasing root osmolarity in Grenache and Semillon grapevines (Rogiers et al., 2011). An initial rise in carbohydrate status under water deficit is consistent with greater reductions in growth than photosynthesis. Hence, carbohydrate storage is likely to decrease under prolonged deficit as a result of higher photosynthesis decline than carbohydrate consumption for the maintenance of cellular survival (respiratory metabolism and osmotic adjustment) (McDowell, 2011). The question of whether deficit irrigation practice applied to high yielding vines in warm climates may negatively impact carbohydrate reserves necessary for longer term vine productivity and sustainability remains to be investigated.

This study was conducted in a warm irrigated region in North West Victoria, Australia, to address the impacts of lighter pruning and deficit irrigation on vine carbohydrate reserve pool and on the resulting vine production. The key knowledge gaps addressed in this paper are (a) the effect of pruning regimes (mechanical, minimal and spur) on vine size parameters, partitioning of soluble sugar and starch in key vegetative tissues at winter dormancy by Cabernet Franc and on trunk starch concentrations during and between seasons in Shiraz, (b) the effect of irrigation treatment (standard, prolonged deficit) on within-day, within-season and between-season changes in key physiological parameters and tissue carbohydrate concentrations and composition in Cabernet Sauvignon, and (c) vine capacity to maintain productivity and adequately ripen the fruits under the different pruning or irrigation treatments.

## Materials and methods

### Experimental details

Three experiments were conducted in commercial vineyards in the Sunraysia region of Victoria, Australia, to address the effects of pruning (Experiment 1 and 2) or water supply (Experiment 3) on vine carbohydrate status, and on yield components and berry sugar content.

Experiment 1 (1986–87) was carried out at the CSIRO Division of Plant Industry Sunraysia site (34°18′S, 142°5′E). Cabernet Franc, clone AC.72.8186, vines were planted in 1972 at a row and vine spacing of 3.0 × 2.4 m in two parallel rows and trained on a 1.3-m high, 0.4-m T-trellis. The soil was a gradational reddish, calcareous, mottled sandy clay loam. Irrigation by overhead sprays was over 9 ML/ha per annum (Clingeleffer and Krake, 1992). Spur pruning (44 two-node spurs) and cane pruned treatments (eight 14-node canes retained in winter) were imposed for 10 years to the vines. In 1982, minimal pruning (almost zero pruning) treatments were imposed on some of these vines and called minimal-spur or minimal-cane. The design of the experiment (Experiment 1) was a split plot including five replicates of spur, minimal-spur or minimal-cane treatments.

Two sites were selected on a commercial vineyard (34°25′S, 142°21′E) for Experiment 2 (2004–2006) *and* Experiment 3 (2002–2005). They were planted in 1994 with Shiraz (12 ha) grafted onto Schwarzmann (Experiment 2), and with own-rooted Cabernet Sauvignon (12 ha) (Experiment 3), at a density of 1366 vines per ha (3 m between row and 2.44 m within row). The soil was a Nookamka sandy loam (Hubble and Crocker, 1941). Vines were trained on a two-wire vertical trellis and mechanically hedged (600–700 buds per vine). The Shiraz site (Experiment 2) also included vines which had been converted to spur (350 buds per vine) and minimal pruning (1450 buds per vine) as part of replicated trials in 2000. Spur, mechanical, and minimal pruning treatments in Experiment 2 were applied *via* a fully randomized block design with eight replications per plot. The vineyards in Experiment 2 and 3 were drip irrigated prior to budburst to bring soil water content to field capacity. Hence, standard drip irrigation (STD) treatment was applied throughout the season to meet 100% of estimated ET_c_. On the Cabernet Sauvignon site (Experiment 3), STD was compared with a Prolonged Deficit (PD) treatment. The STD and PD irrigation treatments were allocated randomly to plots in a block design with 12 replicates. A more detailed description of the irrigation treatments design is provided by Cooley et al. (2006) and Glenn et al. (2010). The PD treatment was implemented during specific phenological stages that were categorized by the Modified E-L system (E-L) (Coombe, 1995). It (PD) consisted of a standard RDI strategy implemented post flowering (E-L 27-28) to the pre-véraison period of berry size ranging from 4 to 6 mm (E-L 29-30), followed by a period of no applied water (extreme deficit) after the RDI strategy E-L 29-34 to véraison. Irrigation supplies for STD and PD treatments were based on neutron-probe data readings (Hydroprobe model 503DR, CPN, Corporation, 2830 Howe Rd, Martinez, CA, USA), with three replicates for mechanical pruning in Experiment 2 and two to three replicates per irrigation treatment in Experiment 3 (data not shown). STD irrigation ranged from 5.5 ML/ha (2004–05) to 5.9 ML/ha (2005–06) for the Shiraz trial (Experiment 2). The respective amounts of irrigation for STD and PD on Cabernet Sauvignon (Experiment 3) were 4.5 and 4.0 ML/ha (2002–03), 6.5 and 4.9 ML/ha (2003–04) and 4.5 and 3.8 ML/ha (2004–05). Soil moisture content (0–70 cm soil profile) on STD treatment of Shiraz was on average 243 mm during season 2004–05, and 220 mm during season 2005–06. For Cabernet Sauvignon, soil moisture content of STD was approximately 290 mm during seasons 2002–03 and 2003–04, and 252 mm during season 2004–05. Minimal soil moisture during PD treatment ranged from 150 mm (season 2002–03) to 200 mm (seasons 2003–04 and 2004–05). Daily maximum and minimum air temperature and vapor pressure were recorded in Experiment 2 and 3 by a weather station controlled by the Australian Government Bureau of Meteorology and located in the region (34°23′S, 142°08′E).

### Plant measurements

In Experiment 1, the size and the carbohydrate status of Cabernet Franc vines, managed under different pruning regimes, were determined at the end of winter dormancy, prior to budburst (Rühl and Clingeleffer, 1993). Five replicate vines per treatment were mechanically removed from well-moistened soil (Clingeleffer and Krake, 1992). Total vine size and the weights of 1-year-wood (mature shoot), old-wood (trunk and cordons), and major root system were measured. In addition, nodes numbers per mature shoots were counted. Samples of these above-ground and root tissues were collected for carbohydrate (reducing sugars, sucrose, and starch) analyses using enzymatic and HPLC techniques as described by Rühl and Clingeleffer (1993).

The effect of pruning or irrigation on trunk starch content was assessed on Shiraz (Experiment 2) and Cabernet Sauvignon (Experiment 3) at winter dormancy pre-budburst, fruit set, véraison, berry ripeness/harvest, and leaf fall. In addition, trunk reducing sugars and sucrose concentrations were assayed at all stages in Experiment 3. Trunk samples were taken approximately mid-way between the graft union and the training wire, using an increment borer. They were collected on six replicate vines for spur and mechanical pruning and four replicate vines for minimal pruning in Experiment 2, and on 12 replicates vines for each water treatment in Experiment 3 (Clingeleffer and Pellegrino, 2006; Cooley et al., 2006). Samples were snap frozen in liquid nitrogen and stored at −70°C before being freeze dried and ground (Coffee ‘n Spice grinder, Breville, NSW, Australia). Their carbohydrates were extracted according to the method of Rühl and Clingeleffer (1993), adding an internal standard, sorbitol (Sigma). The starch in the pellet was determined using a Megazyme (Bray, Co. Wicklow, Ireland) starch analysis kit. Starch was treated with dimethyl sulphoxide (Sigma-Aldrich, St Louis, MO, USA) to remove resistant starch and then broken down into glucose equivalents using α-amylase and amylo-gluconidase. The absorbance of glucose equivalents was measured at 510 nm. The supernatants, containing soluble sugars, were dried down in a water bath (100°C), suspended in MilliQ water (300 μl), and filter using Sephadex G-25-80 (Sigma) columns. Columns were made by activating a solution of Sephadex (twice the volume of 1 M NaOH 1 h), and washing the Sephadex with twice the volume of water until neutral pH was obtained. Activated Sephadex (500 μl) was then packed into a Nanospec 0.45 μm centrifugal device (Pall Life Sciences) and spun at 500 g for 2 min. The samples were added and spun out from the column (500 g for 1 min). A 100 μl aliquot of water was used to wash the column. Sucrose, glucose and fructose were analyzed by HPLC (GBC Scientific Equipment Pty Ltd, Dandenong, Victoria, Australia). The standards sucrose, glucose, fructose, mannitol, and sorbitol were obtained from Sigma. Detection was at 195 nm. Sample injection volume was 20 μl. Sample concentration was determined using peak area. All reagents used in the extraction were HPLC grade and sourced from Sigma-Aldrich Co. (USA).

In the Cabernet Sauvignon trial (Experiment 3), daily changes in photosynthesis (An), stomatal conductance (gs) and leaf temperature (T_L_) were assessed, using a CIRAS-1 Photosynthesis System (PP Systems, Amesbury, MA, USA) on basal leaves opposite to the bunches of 12 plants per irrigation treatment on 1 day in 2003 (119 days after budburst, modified E-L stage 30–31), during the extreme deficit period of the PD treatment. Water potential measurements (Ψ_*l*_) were measured on the same leaves with a pressure chamber (PMS Instruments Model 610, Corvallis, OR). These leaves were then snap frozen in liquid nitrogen, and stored at −70°C until they were assayed for starch concentrations. Leaf samples were ground in liquid nitrogen using a mortar and pestle. Starch analysis was conducted using the Megazyme starch analysis kit, as above for trunk samples (Cooley et al., 2006).

Yields of all treatments of Experiment 1, 2, and 3 were determined by weighing the grape clusters from each vine. Five clusters per vine were randomly collected to subsample 100 berries. The berry sub-samples were weighed, homogenized in a mortar and pestle, filtered through a sieve and the total soluble solids (TSS) concentrations (°Brix) were determined on the berry juice using a temperature compensating digital refractometer (Atago, Tokyo, Japan).

### Statistical analysis

The effects of pruning or irrigation treatments by season or of season on plant size, leaf physiology, tissues carbohydrates, and productivity were assessed with a One-Way analysis of variance. Where significant differences were found, mean values were separated using Fisher's least significant difference (LSD) test (*P* = 0.05).

Data were analyzed using Genstat 5 (Experiment 1) and Genstat 6 (Experiment 3) statistical packages (Genstat 5 or 6 Committee, Numerical Algorithms Group, Oxford, UK), and with R (Experiment 2)—language and environment for statistical computing (R Development Core Team, 2012).

## Results

### Vine size, carbohydrate status, and productivity as influenced by pruning system in cabernet franc (experiment 1)

Total vine size at the end of dormancy in Cabernet Franc was similar for the spur- and minimal-spur treatments which were about 25% larger than the minimal-cane treatment (Table 1). It should be noted that the cane pruned vines were smaller than spur pruned vines before minimal pruning treatments were imposed, presumably due to the removal of both 1- and 2-year wood at pruning. Spur pruning treatment produced significantly higher 1-year-wood and roots compared with minimal-cane pruned vines (Table 1). The weight of old-wood (trunks and cordons) was highest with the minimal-spur treatment and smallest with the minimal cane treatment (Table 1). The spur and minimal-spur treatments, which had similar total vine sizes, matured the same number of nodes.

**Table 1:** **The impact of pruning technique on Cabernet Franc vine size and carbohydrate composition at the end of winter dormancy, and on yield and total soluble sugars (TSS) at harvest, season 1986–87**.

	**Spur**	**Minimal-Spur**	**Minimal-Cane**
Total vine size (kg)	30.2^a^	32.2^a^	24.2^b^
One-year-wood (kg/vine)	3.0^a^	1.0^b^	0.8^c^
Old-wood (kg/vine)	20.4^b^	25.7^a^	18.2^c^
Roots (kg/vine)	6.7^a^	5.6^ab^	5.2^b^
Nodes/vine	1499^a^	1500^a^	1308^b^
Stored carbohydrates (g/vine)	2041^a^	2172^a^	1680^b^
Reducing sugar (g/vine)	347^b^	447^a^	351^b^
Sucrose (g/vine)	105	73	66
Starch (g/vine)	1764^a^	1653^a^	1264^b^
Yield (kg/vine)	18.7	21.2	18.9
TSS (°Brix)	25.7^a^	22.9^b^	23.0^b^
Sugar in berries (g/vine)	4780^a^	4810^a^	4350^b^
Berry/stored carbohydrates	2.34	2.21	2.59

*Letters following means within rows indicate significant differences (P < 0.05) between treatments determined by LSD (P < 0.05). Adapted from Clingeleffer and Krake (1992) and Rühl and Clingeleffer (1993)*.

**Figure 1:**
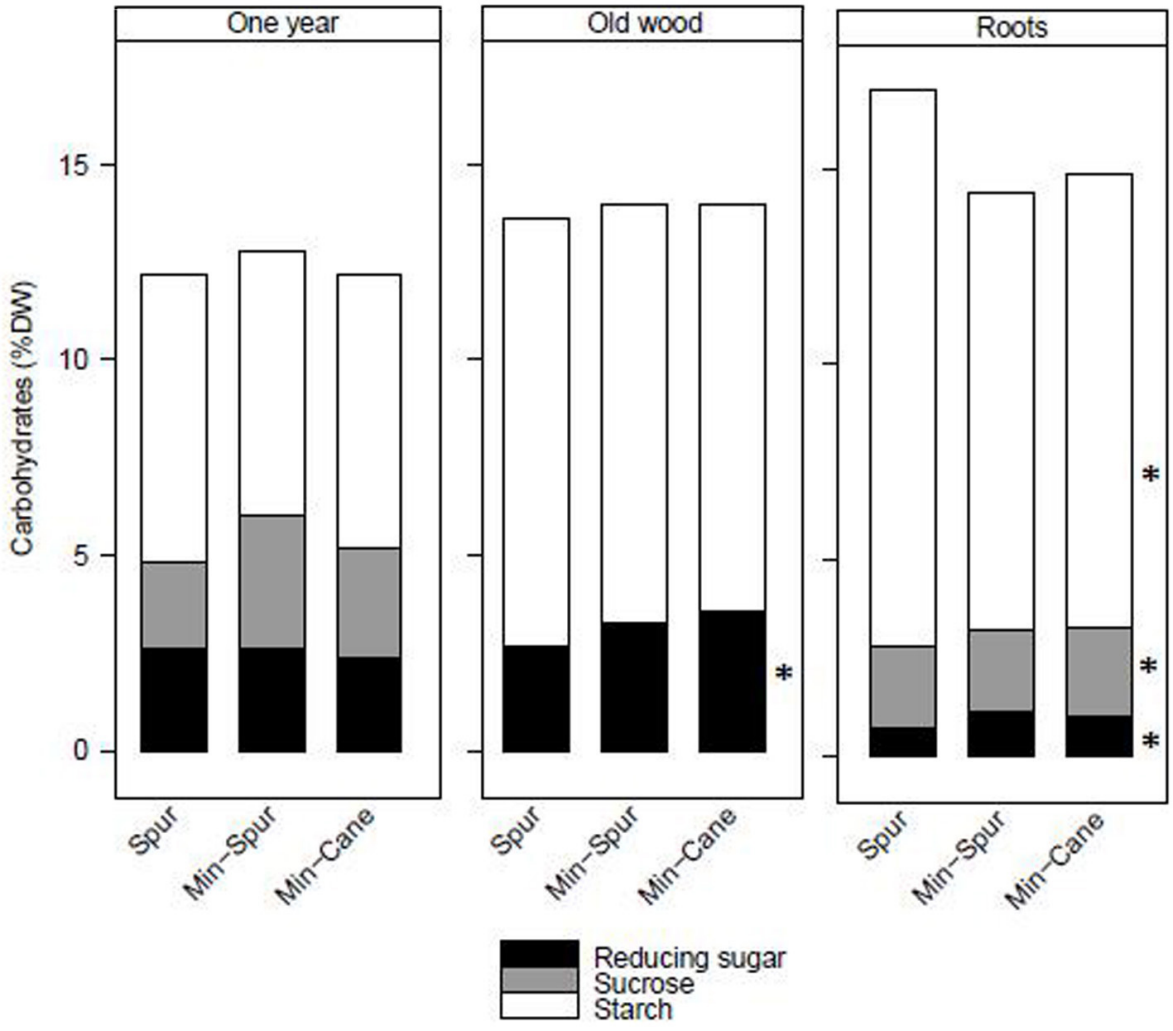
**The effect of pruning technique on starch, sucrose, and reducing sugar concentrations (% dry weight) in plant parts of Cabernet Franc at the end of winter dormancy for season 1986–87**. Within treatment LSD differences (*P* < 0.05) are indicated by an asterisk. Adapted from Rühl and Clingeleffer (1993).

Total stored carbohydrate and starch amounts at the end of dormancy were similar for spur and minimal-spur vines, which were significantly higher than minimal-cane pruned vines (Table 1). Total reducing sugar amount was the highest for minimal-spur vines (Table 1). The total carbohydrate concentrations in dry matter of 1-year-wood (12.4%), old-wood (13.9%), or roots (15.4%) were unaffected by pruning treatment (*P* > 0.05; Figure 1). However there were differences in the carbohydrate fractions in different plant parts. Starch was the major carbohydrate fraction with average concentrations in 1-year-wood, old-wood, and roots of 7.1, 10.7, and 12.3% dw, respectively (Figure 1). Starch concentrations were unaffected by pruning treatment in 1-year-wood and old-wood but were higher in roots with spur pruning compared to minimal-spur or minimal-cane pruning (Figure 1). Reducing sugar concentrations ranged between 0.9% dw for roots and 3.2% dw for old-wood (Figure 1). They were similar between pruning treatment in 1-year-wood (*P* > 0.05) but lower with spur pruning compared to minimal-spur or minimal-cane in old-wood and roots (Figure 1). Sucrose concentrations were unaffected by pruning treatment in 1-year-wood (2.8% dw) and below the level of detection in old-wood (Figure 1). In roots, sucrose concentration (2.1% dw) was slightly higher for minimal-cane pruning (Figure 1).

At harvest, there were no significant effects of pruning system on yield (20 kg per vine on average), but soluble solids were higher with spur pruning (25.7°Brix) than with either minimal pruning treatment (22.9–23°Brix), possibly due to desiccation (Table 1). As a consequence, the total sugar in berries was similar for the spur- and minimal-spur pruned vines but significantly lower for minimal-cane pruned vines (Table 1). No significant effect of pruning on the ratio of berry sugars to the level of carbohydrates stored by the plant during dormancy was observed (Table 1). Sugar in berries was on average 2.4-fold higher than vine stored carbohydrates.

### Carbohydrate seasonal dynamics and productivity as influenced by pruning system in shiraz (experiment 2)

**Figure 2:**
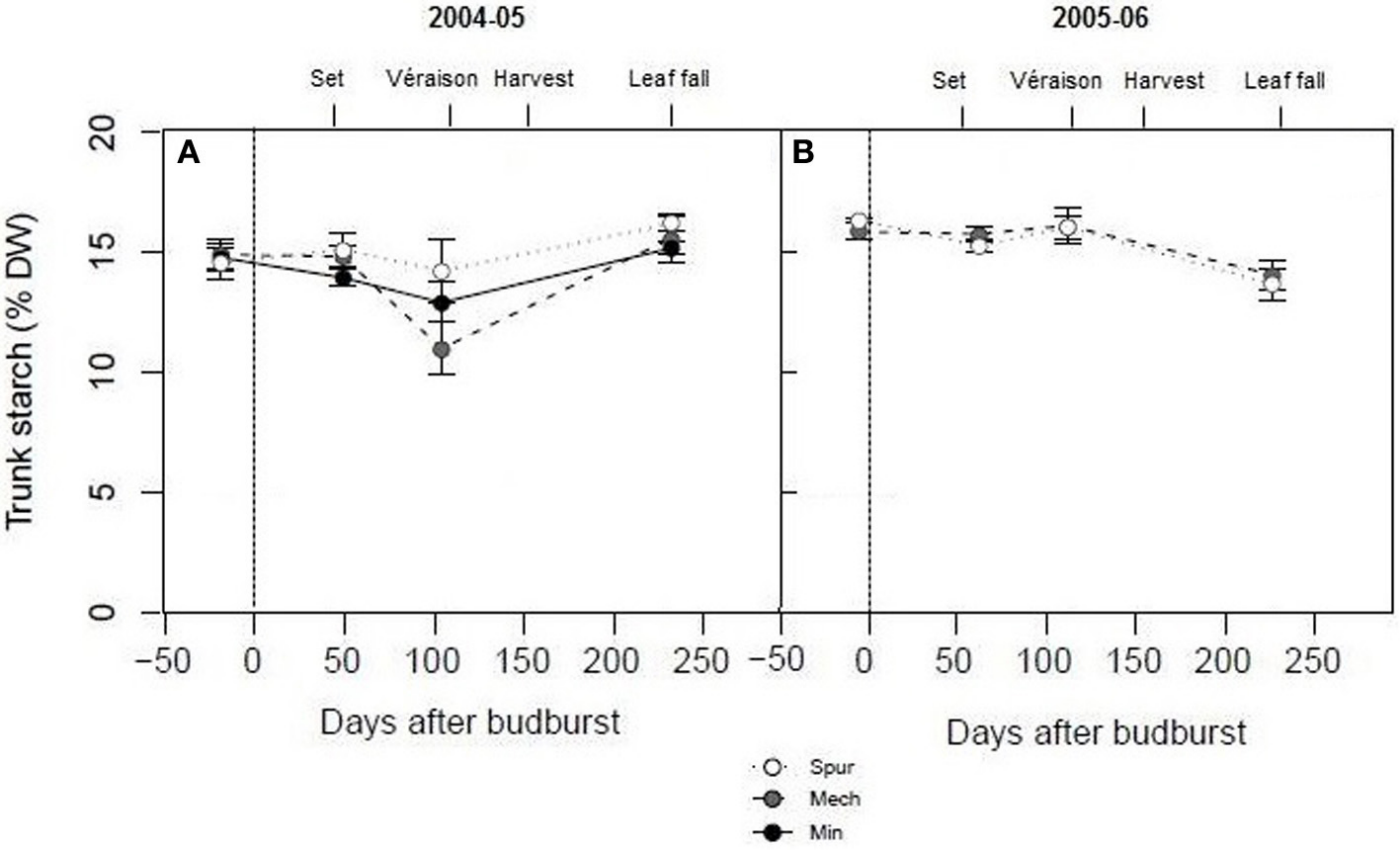
**The effect of pruning technique on Shiraz trunk starch concentration (% dry weight) for seasons 2004–05 (A) and 2005–06 (B)**. The time of budburst (BB) is delimited by a dashed line. Fruit set, véraison, harvest, and leaf fall are indicated. Bars are ± standard error.

In order to assess the effect of spur, mechanical hedging, and minimal pruning on vine carbohydrate dynamics over the cropping season, trunk starch concentrations of Shiraz were determined at key stages of development from budburst to leaf fall. Trunk starch concentrations were quite high (15% dw on average) compared with Experiment 1 at most phenological stages (budburst, set, and leaf fall) and for all pruning treatments (Figure 2). However, significant differences were observed between and within the two seasons. Trunk starch concentration was significantly lower (*P* < 0.05) during season 2004–05 (14.4% dw) compared with season 2005–06 (15.4% dw). The lowest trunk starch concentrations were observed at véraison in 2005 (14.2% dw for spur, 12.9% dw for minimal, and 11% dw for mechanical pruned vines), and at leaf fall in 2006 (14% dw) (Figure 2). Apart from a tendency for mechanical hedging and minimal pruning to have lower trunk starch concentrations than spur pruning at véraison in 2005, the differences between pruning treatments were not significant (*P* > 0.05; Figure 2), similar to the results of Experiment 1.

Yields at harvest were 40% lower in 2005 (mean of 12 kg per vine) compared with 2006 (mean of 20 kg per vine) (*P* < 0.05), partly due to lower berry weight (12% reduction). No seasonal difference in TSS was observed (mean TSS 24.6°Brix) (*P* > 0.05, Table 2). As a result, TSS per berry was lower in 2005 compared with 2006. Yields, berry weight, and TSS per berry at harvest did not differ between the pruning treatments for both seasons. TSS only slightly varied between pruning treatments in 2005, with higher values for minimal pruning (25.7°Brix) than spur and mechanical pruning (24.4°Brix and 24.3°Brix) (Table 2).

**Table 2:** **The effect of pruning technique on Shiraz yield, berry weight, and total soluble sugars (TSS) at harvest, seasons 2004-05 and 2005-06**.

**2004–05**			
	**Spur**	**Mechanical**	**Minimal**
Yield (Kg/vine)	12.5	13.0	10.3
Berry weight (g)	1.31	1.24	1.22
TSS (°Brix)	24.4^b^	24.3^b^	25.7^a^
TSS/berry (g)	0.32	0.30	0.32
**2005-06**			
	**Spur**	**Mechanical**	
Yield (Kg/vine)	18.9	21.3	
Berry weight (g)	1.46	1.41	
TSS (°Brix)	24.7	24.1	
TSS/berry (g)	0.36	0.34	

*Letters following means within rows indicate significant differences (P < 0.05) between treatments determined by LSD*.

### Vine physiology, carbohydrate seasonal and diurnal dynamics and productivity as influenced by deficit irrigation in cabernet sauvignon (experiment 3)

**Figure 3:**
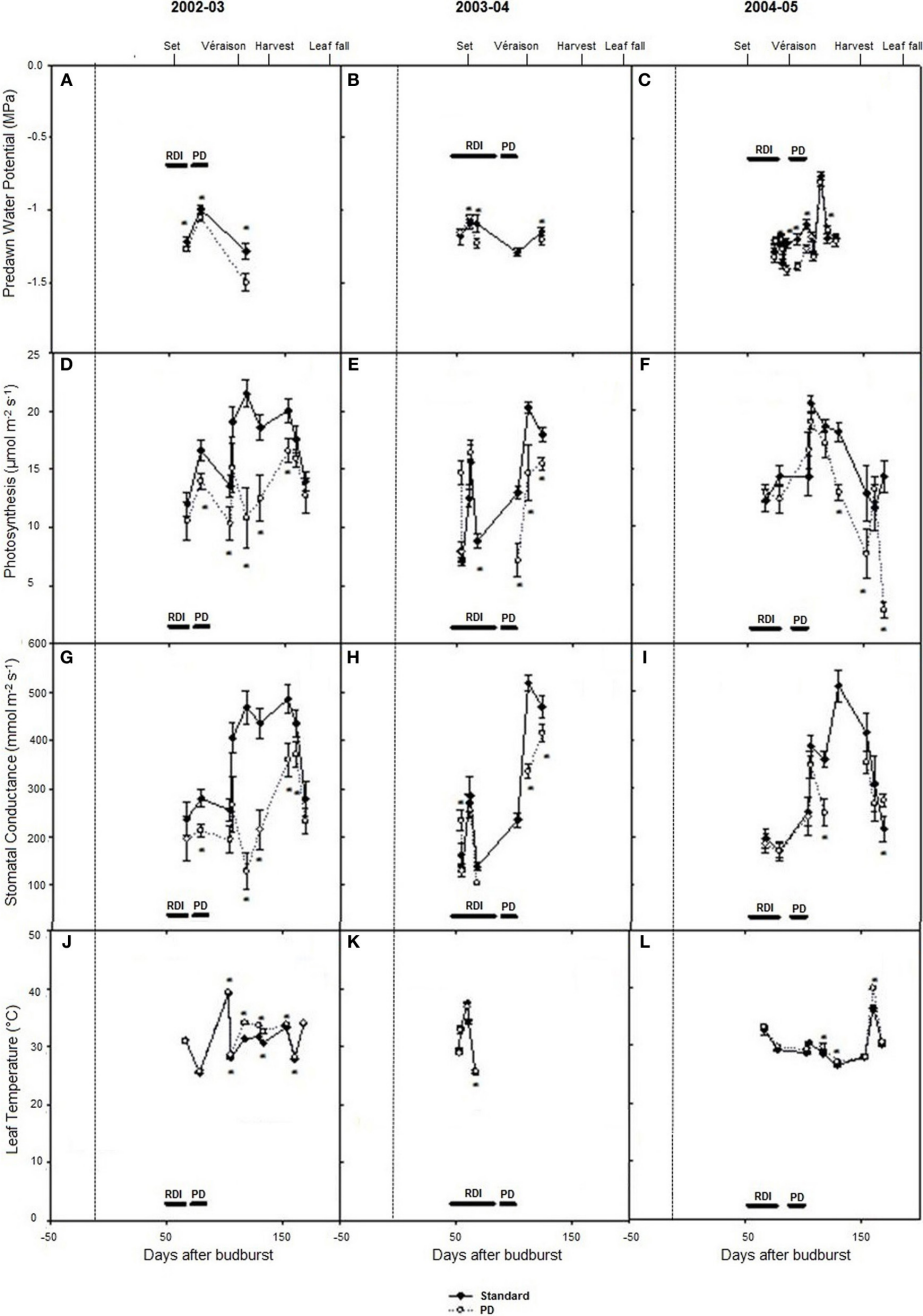
**The effect of irrigation on Cabernet Sauvignon predawn water potential (A–C), photosynthesis (D–F), stomatal conductance (G–I), and leaf temperature (J–L) for seasons 2002–03, 2003–04, and 2004–05, respectively**. Black lines labeled RDI and PD indicate periods of water deficit treatment. The time of budurst (BB) is delimited by a dashed line. Fruit set, véraison, harvest, and leaf fall are indicated. Bars are ± standard error; within treatment LSD differences (*P* < 0.05) are indicated by an asterisk.

Predawn water potentials were more negative during periods of deficit irrigation treatments in season 2002–03, and periodically during seasons 2003–04 and 2004–05 (Figures 3A–C), indicating that deficit treatments (PD) resulted in periods of vine water deficit. The PD treatment decreased photosynthesis (*P* < 0.05) compared with STD (Figures 3D–F), especially in season 2002–03. Reductions in stomatal conductance in 2002–03 (Figure 3G) were observed concurrently with reductions in photosynthesis (Figure 3D) in the PD period, while leaf temperature increases were observed post PD in 2002–03 and 2004–05 (Figures 3J,L).

**Figure 4:**
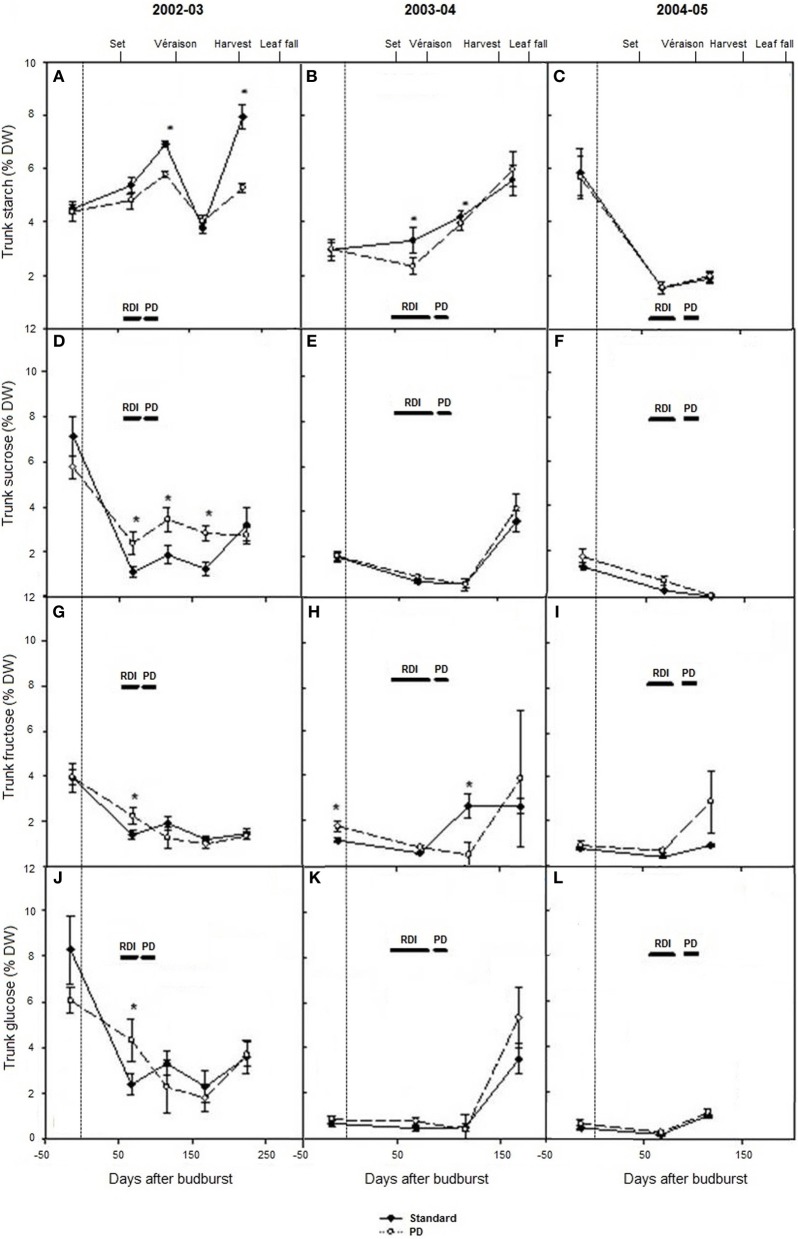
**The effect of irrigation on Cabernet Sauvignon trunk starch concentration (A–C), sucrose concentration (D–F), glucose concentration (G–I), and fructose concentration (J–L) (% dry weight) for seasons 2002–03, 2003–04, and 2004–05, respectively**. Note: 2005 data are missing at leaf fall. Black lines labeled RDI and PD indicate periods of water deficit treatment. The time of budburst (BB) is delimited by a dashed line. Fruit set, véraison, harvest and leaf fall are indicated. Bars are ± standard error; within treatment LSD differences (*P* < 0.05) are indicated by an asterisk.

Seasonal changes in trunk carbohydrates from winter dormancy pre-budburst to leaf fall were measured during seasons 2002–03 to 2004–05 (Figures 4A–L). Trunk starch concentrations were low (1–8% dw) compared with Experiment 1 and 2. Highest concentrations were observed at leaf fall in 2003 and 2004, and lowest levels around véraison in 2005 (Figures 4A–C). Trunk starch concentrations were lower during the early part of the season in 2003–04 relative to 2002–03 (*P* < 0.05), and they were again lower during the véraison period around 100 days after budburst in 2005 (Figures 4A–C). Significant seasonal variations were also found with most soluble trunk carbohydrates measured (sucrose, glucose, and fructose) (Figures 4D–L). Across the seasons, trunk sucrose concentrations ranged between <1 and 7% dw, trunk fructose between 1 and 4% dw, and trunk glucose between <1 and 8% dw (Figures 4D–L). Total soluble carbohydrates and total carbohydrates were significantly higher in 2002–03 than in season 2003–04 and season 2004–05, because of higher concentrations in all soluble carbohydrates measured, in addition to starch (data not shown). Water deficit (PD) decreased starch concentration compared with STD at the time of water stress in 2002–03 and 2003–04 and at leaf fall in 2003 (Figures 4A,B). No differences in starch concentration (*P* > 0.05) were observed pre-budburst during season 2003–04 between STD and PD treatments, suggesting no carry over effect from the stress incurred in season 2002–03. In 2004–05, trunk starch concentration was similar between STD and PD treatments (Figure 4C). The PD treatment maintained or increased trunk sucrose, glucose, and fructose concentrations during the stress period in season 2002–03 (Figures 4D,G,J). A similar pattern was generally observed during most sampling times in seasons 2003–04 and 2004–05 (Figure 4).

**Figure 5:**
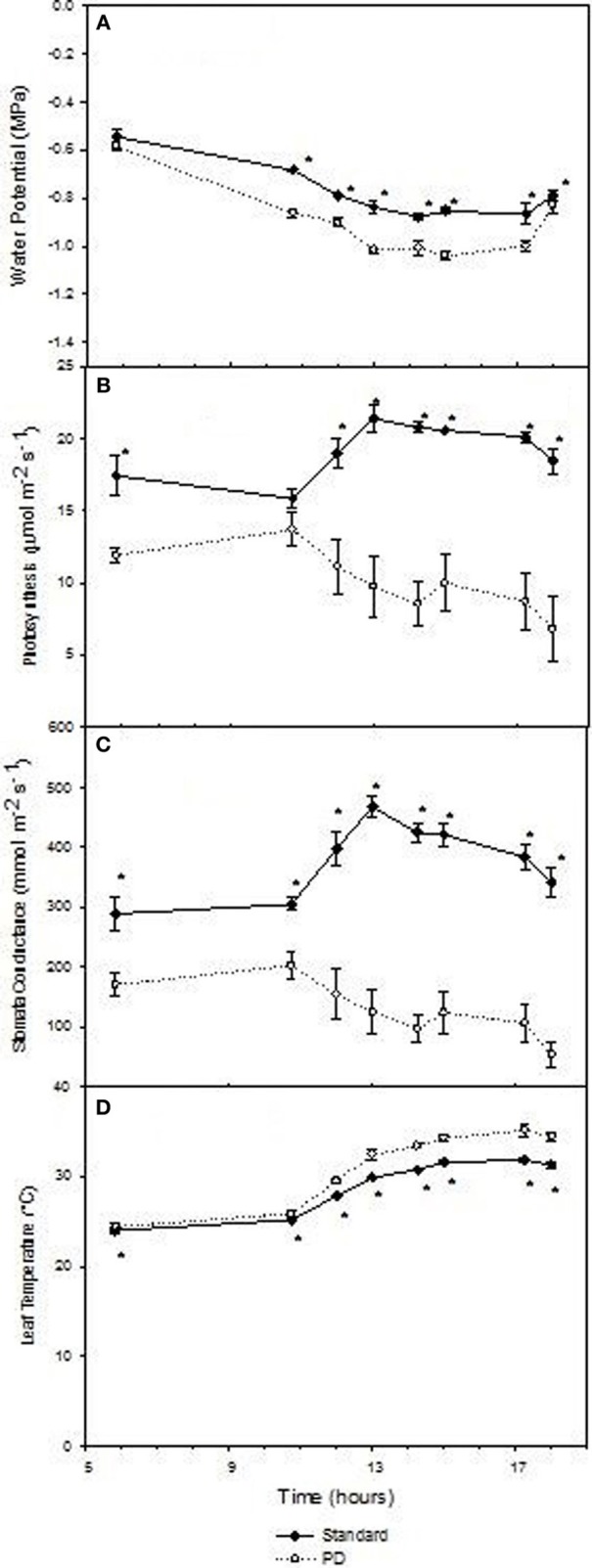
**The effect of irrigation on Cabernet Sauvignon leaf water potential (A), photosynthesis (B), stomatal conductance (C), and leaf temperature (D) throughout a day at the end of the prolonged deficit stress period for season 2002–03**. Bars are ± standard error; within treatment LSD differences (*P* < 0.05) are indicated by an asterisk.

**Figure 6:**
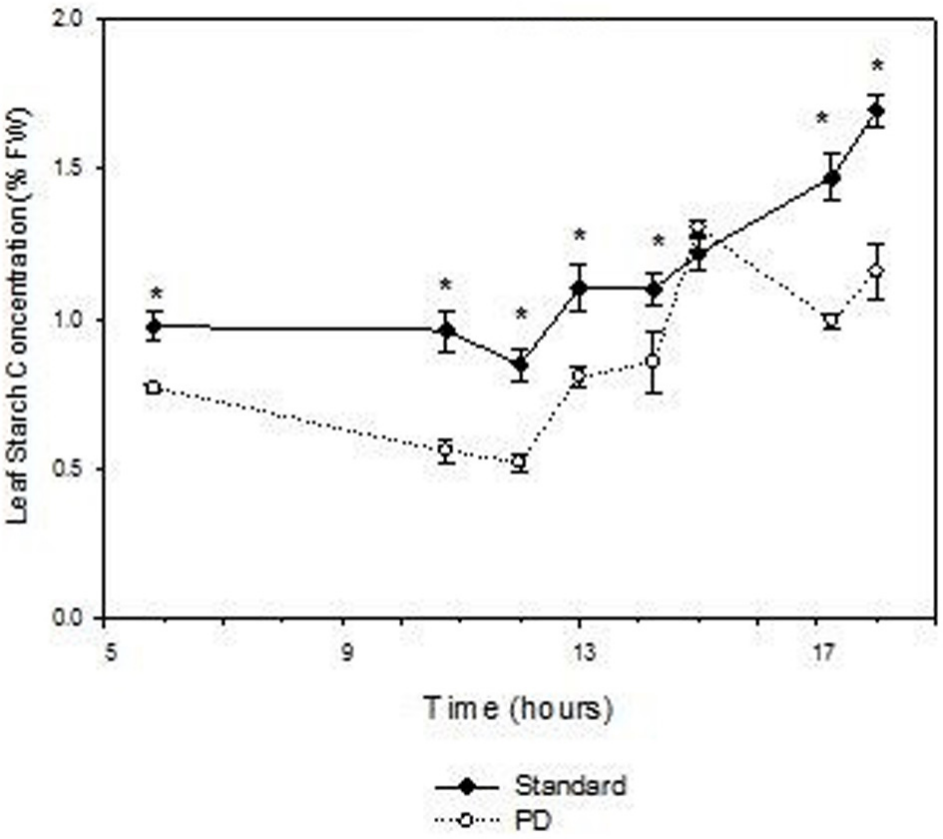
**The effect of irrigation on Cabernet Sauvignon leaf starch concentration (% fresh weight) throughout a day at the end of the prolonged deficit stress period for season 2002–03**. Bars are ± standard error; within treatment LSD differences (*P* < 0.05) are indicated by an asterisk.

Daily effects of deficit irrigation treatments on leaf physiology and starch concentration were studied at the end of the extreme deficit stress (PD) period in 2003, which was pre-véraison or 119 days after budburst. Minimum and maximum air temperatures on this day were 13 and 32°C, respectively, and minimum and maximum VPD were 0.4 and 4 kPa, respectively. Leaf water potential was higher (*P* < 0.05) for STD than PD throughout the day, but not at predawn (*P* > 0.05; Figure 5A). Photosynthetic rate (An) and stomatal conductance (gs) were maximal at solar noon for STD (Figures 5B,C), with values about 22 μmol m^−2^ s^−1^ for An and 460 mmol m^−2^ s^−1^ for gs. Significant reductions in An and gs were found with the PD treatment compared to the STD treatment throughout the day. Maximal values measured in the morning were 14 μmol m^−2^ s^−1^ for An and 200 mmol m^−2^ s^−1^ for gs. The lower gs in PD compared with STD could be associated with higher leaf temperatures (Figure 5D). A 2°C difference in leaf temperature was observed between STD and PD treatments at solar noon. Leaf starch concentration in both the STD and PD treatments (2003) increased from the pre-dawn measurement (0.75% fw for PD and 1.0% fw for STD) to early evening (1.1% fw for PD and 1.7% fw for STD), with lowest values (0.5% fw for PD and 0.8% fw for STD) around mid-day (Figure 6). The PD treatment vines had lower leaf starch concentration at most time points throughout the day compared to the STD (Figure 6).

Significant differences in yield and TSS at harvest were observed between the seasons. Yield was 23 to 34% higher (*P* < 0.05) in season 2004–05 (mean of 19.5 kg per vine) compared to season 2003–04 (mean of 15 kg per vine) and 2002–03 (mean of 13 kg per vine). Berry weight was similar in season 2002–03 and season 2004–05 but significantly lower in season 2003–04 (Table 3). TSS/berry was slightly lower in season 2003–04 compared to seasons 2002–03 and 2004–05 (Table 3). TSS ranged from 24°Brix (STD in 2003 and 2004) to 24.5°Brix (PD, 2003), and 24.7°Brix (STD, 2005). The deficit irrigation treatment (PD) resulted in lower yields in seasons 2002–03 and 2004–05 (16 and 21% reductions, respectively), but not in season 2004 (Table 3). Berry weight and TSS/berry were decreased in PD for all seasons. However, TSS was increased under water deficit (PD) in season 2002–03, but not in 2003–04 and 2004–05 (Table 3).

**Table 3:** **The effect of irrigation treatment on Cabernet Sauvignon yield, berry weight, and total soluble sugars (TSS) at harvest, seasons 2002–03, 2003–04, and 2004–05**.

	**Control**	**PD**
**2002–03**		
Yield (Kg/vine)	14.0^a^	11.7^b^
Berry weight (g)	0.95^a^	0.78^b^
TSS (°Brix)	24.0^a^	24.5^b^
TSS/berry (g)	0.22^a^	0.19^b^
**2003–04**		
Yield (Kg/vine)	15.4	14.7
Berry weight (g)	0.84^a^	0.75^b^
TSS (°Brix)	24.0	24.1
TSS/berry (g)	0.20^a^	0.18^b^
**2004–05**		
Yield (Kg/vine)	21.8^a^	17.2^b^
Berry weight (g)	0.90^a^	0.80^b^
TSS (°Brix)	24.7	24.2
TSS/berry (g)	0.22^a^	0.20^b^

*Letters following means within rows indicate significant differences (P < 0.05) between treatments determined by LSD*.

## Discussion

### Lighter pruning enhanced carbohydrate status through an increase of plant size, but had minor effects on carbohydrate concentrations in vegetative tissues and berries, and on yield

In the pruning studies on Cabernet Franc and Shiraz, treatment effects on the total carbohydrate pool at the end of dormancy were largely due to differences in vine size, rather than differences in carbohydrate concentration or composition in the different vegetative plant organs (Table 1, Figures 1, 2). Trunk starch concentrations were similar between the pruning treatments at all stages of cropping seasons (Figure 2). Carbohydrate concentrations were also little impacted by pruning in all wood fractions of Riesling (Weyand and Schultz, 2006). Similarly to other studies (Smith and Holzapfel, 2009; Holzapfel and Smith, 2012), starch was the major carbohydrate stored in vegetative tissues for all pruning treatments of Cabernet Franc, although significant concentrations of reducing sugars and sucrose were also observed in the above ground tissues, and to a lower extent in roots at the end of dormancy. Soluble sugars represented about 40, 25 and 20% of the carbohydrates in 1-year-wood, old-wood, and roots, respectively (Figure 1). Sommer and Clingeleffer (1996) reported that about two thirds of the carbohydrates in the old-wood and trunks of Cabernet Sauvignon were soluble sugars with reducing sugars and sucrose in equal proportions, compared to about 30% in roots.

The 20% smaller pools of total carbohydrate in minimal-cane pruned vines compared with the spur and minimal-spur vines were mainly due to the development of less old-wood (trunks and cordons) and a smaller root system, thus highlighting the importance of these tissues for carbohydrate storage. Vine vigor was reduced in the minimal-cane treatment through lower pruning weight and node numbers (Table 1). When grafted on high vigor rootstocks, such as Ramsey, Cabernet Sauvignon had higher carbohydrates status prior to winter and higher vine vigor, compared with less vigorous ungrafted Cabernet Sauvignon (Sommer and Clingeleffer, 1996). It has been postulated that differences between spur and cane pruning were due to the depletion of carbohydrates through removal of 1-year-wood and two-year-wood at pruning. According to Rühl and Clingeleffer (1993), removal of 90% of the one-year-wood by spur pruning in Cabernet Franc would reduce the total carbohydrate pool by 8% (i.e., 10% of reducing sugars, 31% of sucrose, 6% of starch). When cane pruned, it is likely that the total carbohydrate pool may be depleted by about 16% due to the removal of 1- and 2-year-wood, thus leading to the development of smaller vines (Clingeleffer and Krake, 1992).

Although minimal-cane pruned vines of Cabernet Franc were 25% smaller than spur and minimal-spur vines, they only produced 10% less total berry sugar (Table 1). Therefore, TSS in berries slightly varied between the pruning treatments in Cabernet Franc, and this was also observed, to a lesser extent, for the pruning study on Shiraz (Tables 1, 2). Carbohydrates accumulated in fruit and removed at harvest were on average 2.4 times higher than the total carbohydrates stored in the dormant vine across all pruning treatments with Cabernet Franc (Table 1), as described in Rühl and Clingeleffer (1993). In the Cabernet Sauvignon rootstock study of Sommer and Clingeleffer (1996), this ratio has been calculated to be 3.0 and 3.5 for minimal and cane pruned vines, respectively. These results indicate that high carbohydrate concentrations are accumulated, and removed at harvest, and that photosynthetic assimilate is partitioned preferentially by fruit. Yield and berry weight were also unaffected by pruning systems in our study (Tables 1, 2). However, vines with a relatively high proportion of old-wood and thus high capacity for carbohydrate storage, as generally observed under minimal pruning, were shown to have higher yields in other studies (Winkler, 1958; Koblet and Perret, 1985; Clingeleffer and Sommer, 1995; Weyand and Schultz, 2006). Simple, non-destructive methods for assessing total vine size and old-wood mass, would thus assist in studies involving the carbohydrate pool and dynamics, and their effects on yield.

### Water deficit reduced starch concentrations and increased concentrations of soluble sugars in vegetative tissues, lowered yield, but slightly influenced concentrations of sugars in berries

Significant within season variations in trunk carbohydrate were observed on Cabernet Sauvignon, and to a lower extent on Shiraz (Figures 2, 4). As reported in other studies (Winkler, 1958; Bains et al., 1981; Zapata et al., 2004; Holzapfel and Smith, 2012; Zufferey et al., 2012), carbohydrate concentrations were generally high in winter, with a marked decline, especially of soluble sugars, during spring as canopies developed, and an increase toward the end of the growth period. The proportions of soluble sugars in wood were high at dormancy and at leaf fall in the Cabernet Sauvignon and Cabernet Franc trials, suggesting they play a key role in carbohydrate transport from the roots or from the canopy to the woody parts (Figures 1, 4). Vines commenced redirection of carbohydrate assimilate toward storage organs after véraison. The low levels of trunk carbohydrates concentrations between fruit-set and véraison (Figures 2, 4), indicates that this period is critical to carbohydrate partitioning between storage organs and the developing fruit.

Water deficit reduced leaf starch concentration over the day and trunk starch concentrations over the season in Cabernet Sauvignon (Figures 4, 6). These results are in accordance with those of Holzapfel et al. (2010), who reported a decrease in starch concentration in roots and to a lesser extent in wood tissue under water deficit. The decline in trunk and leaf starch concentrations in PD were concomitant with relative reductions in net photosynthesis of up to 3-fold, both at seasonal and daily time steps (Figures 3, 5). For the same experiment, but in other seasons (2003 and 2004), Glenn et al. (2010) observed similar reductions in An and gs for PD during and at the end of the stress period. Leaf temperature significantly increased in PD as a result of stomatal closure (Figures 3J–L). The negative impact of water deficit on carbon balance is likely to be exacerbated under warm climate regions such as Sunraysia in Australia. The use of irrigation to prevent excessive canopy temperature should thus be an important consideration. Water deficit reduced trunk starch recovery after véraison, and trunk total carbohydrates concentration at harvest during season 2002–03 (Figure 4A). As suggested by Smith and Holzapfel (2009), the post-harvest period is fundamental for the replenishment of carbohydrate reserves and the longevity of high yielding grapevines grown under warm climates, especially under limited irrigation. It should be noted that water deficit tended to enhance trunk soluble sugar concentrations (Figure 4). Such higher proportions of soluble sugars were possibly due to an increase in carbon reserve mobilization, as observed in rice under water restriction (Yang et al., 2002) or in defoliated grapevine (Candolfi-Vasconcelos et al., 1994). Starch conversion to soluble sugars in roots under water deficit was also reported to be part of osmotic protection in grapevine, thus maintaining cell turgor (Rogiers et al., 2011).

In addition to the changes in starch and soluble sugar concentrations in vegetative tissues, water deficit also lowered berry size and vine yield, with maximal reductions of 18 and 21%, respectively (Table 3). However, berries TSS were little impacted by water deficit. A very low influence of water deficit on sugar concentration in the berries was also reported by Ojeda et al. (2002).

### Carbohydrate concentrations in vegetative tissues and yield varied widely between varieties and seasons, but sugar concentrations in berries were similar

Over the experiments, there were high varietal differences in carbohydrate concentrations in vegetative tissues (starch and soluble sugars) when compared at dormancy and throughout the season. Trunk carbohydrate status of Cabernet Sauvignon and Shiraz differed during season 2004–05. Vines of the varieties were similarly managed (mechanical pruning and standard irrigation) at the same site. Trunk starch concentrations of Shiraz were very high at all stages of growth (i.e., 12–16% dry matter), compared to Cabernet Sauvignon, where maximum levels at dormancy were around 6% dw in 2004–05 and dropped to values lower than 2% dw at the commencement of véraison (Figures 2, 4). It should be noted that trunk starch concentrations of Chardonnay grafted on Schwarzmann and grown in the same vineyard reached intermediate values during season 2004–05 of 9% dw, and only dropped to 7% dw at véraison. In these comparisons, crop load may have been a confounding factor of starch variations, as yields of Shiraz, Cabernet Sauvignon, and Chardonnay were 13, 22, and 21 kg vine^−1^, respectively in 2005 (Tables 2, 3). When vines were managed similarly on the same CSIRO site (Rühl and Clingeleffer, 1993; Sommer and Clingeleffer, 1996), carbohydrate concentrations were also lower at dormancy in all vegetative tissues of Cabernet Sauvignon (<10% dry matter) compared to Cabernet Franc (12% dry matter in 1-year-wood to and 15% dry matter in roots; Figure 1). While these differences may be due to differences between the varieties, the results may also be confounded by differences between crop loads or seasons because yield was higher for Cabernet Sauvignon compared with Cabernet Franc in the above studies.

Yield markedly varied between seasons in Shiraz and Cabernet Sauvignon under control irrigation (Tables 2, 3). In contrast, leaf area index only slightly differed (data not shown). For Cabernet Sauvignon, maximal leaf area index at véraison for STD reached approximately 3.9 during season 2003–04; and 4.2 during season 2004–05. For the Shiraz trial, leaf area index at flowering ranged from 2 to 2.8 during season 2004–05 and from 2.7 to 3 during season 2005–06 for all pruning treatments. As a result, different source sink ratios across seasons and different degrees of water stress are incurred. When yields were the highest (in 2005–06 for Shiraz and in 2004–05 for Cabernet Sauvignon), mean soil moisture content over the cropping season was 23 to 38 mm lower (0–70 cm soil profile) on Shiraz and Cabernet Sauvignon, and trunk carbohydrate concentrations after véraison were reduced (Figures 2, 4). Higher crop loads also increased water deficit in peach trees (Berman and DeJong, 1996). For the Cabernet Sauvignon trial, the PD treatment resulted in similar leaf area index reduction (of about 0.5) at véraison compared with STD during season 2003–04 and 2004–05 (data not shown). However, the minimal soil water content reached on PD was 50 mm lower during season 2002–03 compared with 2003–04 and 2004–05, and PD differed from STD only after an extended RDI period during season 2003–04 (data not shown). This may explain the higher reduction of source activity (photosynthetic rate) in PD across the season 2002–03, together with the higher decrease in sink reserves (trunk starch) at leaf fall during that season. As a result, yields were lower in PD during seasons 2002–03 and 2004–05. Within and between season fluctuations of other environmental factors, including warm temperature and high VPD may also have differentially impacted carbon balance decline through their negative effects on gas exchange and carbohydrate synthesis (Schultz, 2000; Soar et al., 2006; Flexas et al., 2009; Zufferey et al., 2012). Climate, over the period from budburst to harvest, was slightly more favorable in 2004–05 compared with other seasons (2002–03, 2003–04, and 2005–06), because of cooler temperatures and lower VPD. The upper quartiles of maximum temperature and mean VPD were 32.8°C and 1.6 kPa, respectively in 2004–05, while they ranged between 33.7–34.5°C and 1.8–2.1 kPa, respectively for all other seasons (2002–03, 2003–04, and 2005–06). Thus, source activities and sink reserves were probably impacted by climatic factors to a lower extent in 2004–05 compared with other seasons.

In spite of the large varietal and seasonal differences in yield and carbohydrate concentrations in vegetative tissues, the capacity of plants to ripen berries was remarkably stable in our experiments. TSS in berries only varied between 24.0°Brix and 25.7°Brix for Shiraz and Cabernet Sauvignon, irrespective of years and pruning and irrigation treatments.

## Conclusions

The impacts of lighter pruning and deficit irrigation techniques on carbohydrate dynamics and vine production were addressed in this study for warm irrigated Australian vineyards. The total carbohydrate pool in grapevines was shown to be largely dependent on the size of the vine, mainly determined from old wood and roots system, rather than on the carbohydrate concentration in vegetative tissues. Starch was the major carbohydrate in all vegetative tissues. Soluble sugars also significantly contributed to the total carbohydrate pool in the above ground tissues, and to a lower extent in roots. Carbohydrate composition varied between tissue types, but was similar for all pruning treatments. Large varietal differences in carbohydrate pools were observed, although they were possibly confounded by crop load effects.

Within seasons, the lowest concentrations of stored carbohydrates in woody trunks occurred between fruit set and véraison period, indicating that this period was critical for carbon balance. Water deficit tended to lower recovery of trunk carbohydrates post-véraison and worsen carbon starvation through its negative impact on starch concentrations. Deficit irrigation practices had the potential to impact on source activity, sink reserves, and yield in grapevines. These responses were subject to significant season to season variation which may be due to differences in the response of sink reserves to the water stress and crop load which may then impact on the source reserves. Alternatively, there may be interactions occurring between high ambient temperatures, high VPD and soil water deficit, which significantly affect the canopy's ability to produce carbohydrate and partition it according to sink demand.

Despite the large variations in carbohydrate pool in vegetative tissues, and also the variation in yield components, berries accumulated sugars at a similar rate for all pruning systems, irrigations levels and seasons.

### Conflict of interest statement

The authors declare that the research was conducted in the absence of any commercial or financial relationships that could be construed as a potential conflict of interest.
